# New Insight into the Quality Traits of Milk and Cheese from Teramana Goats, a Native Italian Breed

**DOI:** 10.3390/ani13081344

**Published:** 2023-04-13

**Authors:** Marco Florio, Costanza Cimini, Andrea Ianni, Francesca Bennato, Lisa Grotta, Luca Valbonetti, Giuseppe Martino

**Affiliations:** 1 Department of Biosciences and Technology for Food, Agriculture and Environment, University of Teramo, 64100 Teramo, Italy; 2 Institute of Biochemistry and Cell Biology (CNRIBBC/EMMA/Infrafrontier/IMPC), National Research Council, 00015 Rome, Italy

**Keywords:** biodiversity, Teramana goat, cheese quality

## Abstract

**Simple Summary:**

Enhancement strategies for native goat breeds are essential for sustainable production. In the present study, milk and cheese derived from the Teramana goat, a native bread of the Abruzzo region, were analysed and compared with those of the intensive Saanen breed. The outcomes showed that the milk and cheese of the Teramana were richer in some fatty acids, mainly linoleic acid. A low concentration of compounds such as ketones and esters, and a higher concentration of carboxylic acids found in Teramana cheeses improve the cheese’s oxidative stability during ripening. The variations in volatile profile, lipolytic action and technological characteristics were confirmed by sensory analyses. Our findings open new prospects for the valorisation and preservation of the Teramana goat.

**Abstract:**

The preservation and enhancement of native breeds is a central issue to initiate new breeding policies, which are sustainable and adapted to climate changes. The aim of this study was the characterisation of the qualitative traits of milk and cheese obtained from Teramana goats compared with Saanen goats reared in the same breeding facilities or environment. The research involved 41 Teramana goats and 40 Saanen goats. The milk of each group was collected and used to produce cheese, which was analysed fresh and after 30 and 60 days of ripening. Cheese samples were subjected to evaluations of the physical parameters, including colour and the TPA test, in addition to chemical evaluations that were focused on the determination of total lipids, fatty acids composition, volatile profile and proteolysis. The results showed the Teramana goat to be rich in fat, characterised by a significant increase in conjugates of linoleic acid (CLA), which are attributed to important health benefits. The analysis of volatile compounds showed more oxidative stability of Teramana goats’ cheeses during the ripening. The results from sensory analyses indicated an improved hardness and yellowness, which could be accompanied by an improvement in customer acceptance. In conclusion, our study shows interesting results regarding the milk and cheese from the Teramana goat, as well as a positive evaluation by consumers, findings that encourage the importance of promoting native breeds.

## 1. Introduction

Nowadays, goat milk has been re-appreciated by consumers, in terms not only of milk taste but also of health benefits. Consumers associate goat milk with natural, rural and sustainable farming. The welfare of animal farms is among the major consumer worries, as are also the quality and safety of food products, and the effect of agriculture on climate change [1]. This explains the recent high demand for and consumption of healthy foods in developed countries, as well as the increased interest in dairy goats and goat milk products [2]. Goat milk is the third most produced variety of milk in the world, after cattle and buffalo, according to statistics from the Food and Agriculture Organisation of the United Nations (FAO). In 2016, about one billion goats were bred with an average milk production of 15,262,116 tons. France has the highest production of goat’s milk in Europe, followed by Spain and Greece [3]. In recent decades, goat milk has doubled its production, with market trends suggesting a further increase of 53% by 2030 [4]. 

Goat’s milk is mainly used to produce dairy products. The quality of the milk, under the technological-dairy profile, is understood as the ability to obtain a good cheese, with an excellent yield [5,6]. In fact, in the last years, there has been an increased interest in the genetic value of rustic goat breeds, with Italy having a potential leading role in this due to its high concentration of native goat breeds [7,8,9]. One of them is autochthonous from Teramo’s province in the Abruzzo region, an endangered goat breed called Teramana goat. This goat was previously described by Ianni et al. [10] as a medium-sized goat with a dark coat (mainly black or dark brown), sometimes presenting with white streaks on the head. The head is long with a straight frontal-nasal profile, with the possibility of having horns in both sexes (see Supplementary Materials). Their structural and anatomical features allow them to exploit the inaccessible and uncomfortable pastures typical of Abruzzo [11]. This Italian region offers several opportunities, such as being a territory useful for sustainable breeding. 

In recent years, breeding policies have focused on intensive goat breeds and focused only on product quantity. However, it is important to consider the advantages of native breeds, with phenotypes linked to the climate and geography of the region in which they evolved, and thus the possibility to acquire higher quality production (both nutritionally and organoleptically) than non-native breeds [12]. Local breeds can produce milk with higher fat and protein content than intensive breeds, characteristics much more appreciated by cheesemakers using goat milk to produce cheese [2,13]. Flavour cheese is related to milk quality, processing operations and microbial activity [14]. The obtained cheese is characterised by a complexity of microbial populations, which contribute to numerous biochemical reactions leading to the formation of volatile compounds [15]. The combination of volatile compounds and their interactions contributes primarily to the formation of the aroma and taste, which together constitute cheese flavour [16,17]. This last aspect, in combination with the overall appearance of cheese and its texture, is decisive for consumer selection and preference [18,19]. Consequently, the importance of studying cheese flavour is related principally to the acceptance of cheese within the market [14]. 

In the current study, we aimed to evaluate the production of cheese from milk derived from the Teramana goat. In order to improve the current knowledge of local breed production, the cheese product was studied along the various stages of maturation, comparing it with that produced by the Saanen goat, a more industrial breed.

A multiple-step approach was adopted to evaluate the potential effects of breed on the qualitative attributes of cheese in terms of total lipids, fatty acid composition, volatile profile and proteolysis. Finally, a sensory analysis was performed to assess the customer acceptance of the products.

## 2. Materials and Methods

The experimental plan was carried out in compliance with Directive 2010/63/EU of the European Parliament (European Union, 2011) and Directive 86/609/EEC (European Economic Community, 1986), relating to the protection of animals used for scientific purposes. The trial was carried out on a dairy goat farm where other livestock practices were introduced in addition to those already adopted by the farmer. Therefore, there were no ethical issues to deal with.

### 2.1. Experimental Design, Cheesemaking and Sampling

This study is part of a project studying the quality of milk and derived dairy products of an endangered goat breed, the Teramana goat, compared with a cosmopolitan breed, the Saanen goat. The study was conducted between February and June 2022. Teramana goats are an endangered breed; forty samples of Saanen goats were collected from the same farms or in the same area as the Teramo goats. At the time of sampling, all animals were homogeneous in terms of lactation period and body weight. In addition, all feed information was collected through questionnaires completed by farmers. The modules required animal feed, including the pasture’s composition and the barn’s type of feed. On all farms, the components of diets were almost similar, considering Abruzzo’s small reality. As a result, the effect of the diet was reduced. Individual milk samples were collected and used to evaluate the milk’s chemical composition and fatty acid profiles. The remaining milk was then pooled and used in cheese production according to the protocol outlined below. Bulk milk was pasteurised at 72 °C for 20 s, and cooled to 40 ± 1 °C. Then, the milk was transferred to a container in which a combination of thermophilic and mesophilic starter cultures was added (500 U/5000 L; White Daily, Chr Hansen, Hoersholm, Denmark). Subsequently, for acidification, rennet was added to the milk at a ratio of 1:20,000 (75% of chymosin and 25% of pepsin; Clerici, Cadorago, Italy). After 45 min of incubation, the curd was broken into small pieces, portioned in aliquots of ca. 3 kg in plastic moulds, and kept at 48 °C ± 1.5 °C until the pH reached 5.20 ± 0.2. At this time, all forms were salted with a solution of NaCl 20% in water and then stored in the ripening room with controlled temperature and humidity (10 °C ± 0.5 °C; 85%, respectively). From the cheesemaking, 6 forms of cheese for each breed were produced, 2 were analysed after 1 (T0), 2 after 30 (T30) and 2 after 60 (T60) days of ripening (see Supplementary Materials).

### 2.2. Fatty Acid Profile of Milk and Cheese

The milk fatty acid profile was extracted according to the official AOAC method [20], while cheese fatty acid was extracted according to the Folch method [21]. For both milk and cheese, 50 mg of extracted lipids were weighed and reconstituted with 1 mL of hexane and methylated with 500 μL of 2N sodium methoxide in methanol. The detection of the fatty acids methyl esters (FAME) in each sample was achieved by following the procedure previously used and described by Florio et al. [22]. The identified fatty acids were reported as a mean relative percentage of the total FAME. The value attributed to each fatty acid was also used to calculate the sum of saturated fatty acids (SFA), monounsaturated fatty acids (MUFA) and polyunsaturated fatty acids (PUFA). Desaturation indices (DI) for C14, C16 and C18 were calculated using the formula suggested by Brogna et al. [23].

### 2.3. Western Blot Analysis of Δ^9^-Desaturase and Fatty Acid Synthase in Somatic Cells

Goat milk samples were centrifugated at 4000× *g* for 20 min at 4° C. The pellet was washed in PBS + EDTA (0.05 M) by centrifugation at 12,000× *g* for 5 min at 4 °C. The cells were resuspended in a Lysis Buffer pH 8.5 (7 M Urea, 2 M Thiourea, 30 mM Tris-HCl, 4% SDS pH 8.5). The extracted proteins were then quantified using the Bradford method [24], using BSA as a standard.

To evaluate the identification of Δ^9^-desaturase and fatty acid synthase, we used Western blot analysis (WB). Protein samples (25 μg) were migrated on an SDS-PAGE 10% gradient gel and blotted on a polyvinylidene difluoride (PVDF) membrane. The membranes were stained with Ponceau solution. Membranes were blocked overnight at 4° C, using TBS-T (10 mM Tris-HCl pH 7.4, 150 mM NaCl, 1% Tween 20). Then, the incubation with primary antibodies was performed for 1 h at room temperature for the identification of Δ^9^-desaturase (1:1000) (SCD1 antibody; Biorbyt Ltd., Cambridge, UK). After washing, membranes were incubated with secondary antibody IgG (1:5000) (donkey anti-rabbit antibody HRP; Biorbyt Ltd., Cambridge, UK). Peroxidase was revealed using the chemiluminescent (ECL) system (WESTAR ηC Ultra 2.0; Bologna, Italy) and the images were digitally captured using an Azure C300 (Chemiluminescent Western Blot Imaging System, Azure Biosystems).

Fatty acid synthase was assessed on the same membranes by stripping the previous antibodies with Restore^TM^ Western Blot Stripping Buffer (ThermoFisher). After washing, membranes were blocked with TBS-T (10 mM Tris-HCl pH 7.4, 150 mM NaCl, 1% Tween 20) for 1 h and incubated with fatty acid synthase antibody (1:1000) (Fatty Acid Synthase antibody; Biorbyt Ltd., Cambridge, UK) in PBS-T for 1.5 h. After washing, membranes were finally incubated with the secondary antibody IgG (1:5000) (donkey anti-rabbit antibody HRP; Biorbyt Ltd., Cambridge, UK) for 1 h and revealed as described previously in this section. At least three biological replicates were performed for each antibody and experimental group.

### 2.4. Cheese Moisture and Total Lipids Content

The cheese lipid fraction was extracted following the AOAC official method [25]. The moisture content was determined according to AOAC methods [20]. The lipid extraction was carried out via acid hydrolysis. Briefly, 3 g of cheese were homogenized with 20 mL of HCl 25% in water and, after deproteinization, lipids were extracted with 80 mL of diethyl ether and petroleum ether (1:1). The upper solution containing non-saponifiable lipids was recovered in a previously calibrated round-bottom flask and the solvent was evaporated to dryness at 38 °C with a Strike-Rotating Evaporator (Steroglass S.r.l, Perugia, Italy). The flasks were then placed in a stove at 50 °C for 1 h to then be left to cool at room temperature. This allowed us to weigh the collected fat which was then expressed as a percentage on a dry matter basis.

### 2.5. Cheese Protein Extraction and Sodium Dodecyl Sulfate Polyacrylamide Gel Electrophoresis (SDS-PAGE)

For cheese protein extraction, the protocol was previously reported by Bennato et al. [26]. The extracted proteins were then quantified using the Bradford method [24], using BSA as a standard. Protein extracts were mixed with an equal volume of sample buffer (0.5 M Tris-HCl, pH 6.8; 2% (wt/vol) SDS; 7% (vol/vol) glycerol; 4.3% (vol/vol) β-mercaptoethanol; 0.0025% (wt/vol) bromophenol blue). The mixture was boiled for 3 min to inactivate the enzymes and denaturate the proteins. The samples (7.5 μg) were loaded onto 12% SDS-PAGE for separation. The electrophoresis was performed in a mini protean III dual slab cell (Bio-Rad Laboratories, Watford, UK) at a constant voltage of 120 V. At the end of the run, the results were visualised by staining the gels with 0.5% (wt/vol) Coomassie Blue R-250, dissolved in 40% (vol/vol) ethanol and 10% acetic acid for 30 min. The densitometric analysis was performed using ImageJ software (National Institutes of Health, Bethesda, MD, USA). The relative caseins, β-lactoglobulin and α-lactalbumin were expressed as a percentage of the total protein content, calculated with the reference lane.

### 2.6. Cheese Volatile Profile

Volatile compounds (VOCs) present in cheese at 60 days of ripening were extracted by solid-phase microextraction (SPME) and the separation was carried out by gas chromatography (GC Clarus 580; Perkin Elmer, Waltham, MA, USA) coupled with a mass spectrometer (SQ8S, Perkin Elmer). The GC was equipped with an Elite-5MS column (length × internal diameter: 30 × 0.25 mm; film thickness: 0.25 µm; Perkin Elmer). For all samples, 4 g of earlier shredded cheese was mixed with 10 mL of saturated NaCl solution (360 g/L) containing the internal standard (4-methyl-2-heptanone). VOCs were extracted using a divinyl-benzene-carboxen-polydimethylsiloxane SPME fibre (length: 1 cm; film thickness: 50/30 215 μm; Supelco) at 60 °C with a 40 min exposition time. Helium was used as a carrier gas at a flow rate of 1 mL/min, and VOCs identification was performed by comparison with the mass spectra of a library database (NIST Mass Spectral library, Search Program version 2.0, National Institute of Standards and Technology, US Department of Commerce, Gaithersburg, MD, USA) and by comparing the eluting order with Kovats indices. Data were expressed as the mean relative percentage of the total VOCs identified.

### 2.7. Evaluation of Colour and Textural Properties

The cheese colour in all the ripening times (T0, T30, and T60) was performed considering the recommendations of the International Commission on Illumination (Commission Internationale de L’éclairage (CIE), 1978), using the colorimeter Konica Minolta Chroma Meter CR-5. Cheese moulds were specifically cut into two halves and colour evaluations were conducted at various points on the inner surface of the cheese. The aperture size of the optical system was adjusted to 3 mm and the samples were characterised for lightness (L*) and yellow/blue chromaticity (b*). The yellow index (YI) was also calculated by using the following formula: YI = 142.86·b*/L*.

Textural properties were evaluated at room temperature (21 ± 1 °C) using an Instron Universal Testing Machine (Model 4452, Instron Ltd., Wycombe, UK) equipped with a 500 N load cell. A TPA test was carried out at a crosshead speed of 0.42 mm s^−1^. Each sample (15 mm × 10 mm × 10 mm) was compressed by 20% of its initial height, using a plunger with a plane, circular surface (58 mm diameter) and employing a double-compression method with a 5 s delay between the first and the second bite. Hardness, cohesiveness and gumminess were determined according to Bourne [27]. The data reported are the average of ten replicates.

### 2.8. Confocal Analysis of Cheese

The microstructure of cheese samples, ripened for 0, 30 and 60 days, was analysed using a Nikon A1r laser confocal scanning microscope, equipped with a Plan Apo λ 60X Oil objective, detector Galvano, with a pinhole size of 69 μm and a pixel size of 0.04 µm. The samples were cut using a blade and the proteins were stained with 0.2% Fast Green, while fats were stained with 0.5% Nile Red diluted in distilled water. We used an average of 2 modes in channels series, as follows: channel 1: Nile Red: λ_exc_ = 488 nm, λ_em_ = 563 nm; channel 2: Fast Green: λ_exc_ = 633 nm, λ_em_ = 650 nm.

### 2.9. Sensory Analysis

A sensory analysis of T60 cheeses was performed by a panel composed of fifteen healthy, non-smoker, non-colour-blinded, untrained panellists (ISO, 4120:2004). Before the analysis, brief training was carried out to verify and discuss the vocabulary. One visual attribute (yellow colour intensity), one odour attribute (odour intensity), four texture attributes (elasticity, hardness, gumminess, creaminess/graininess), five taste attributes (saltiness, sourness, bitterness, sweetness, pungency) and five flavour attributes (mouldy, fruity, rind, hay, grassy) were used to describe the samples. Pagliarini et al. [28] and Bérodier et al. [29] introduced the terms flavour and textural attributes, respectively. The sensory analysis was conducted in a laboratory designed according to ISO 858929 (1985) and equipped with individual cabins and white lighting (D65). Sensory evaluation was performed in two sessions, morning and afternoon, by ISO 6658 (1988). All the samples were evaluated in each session. Samples were equilibrated at room temperature (21.1 °C) before testing and each wheel of cheese was split into two pieces. One half was used ‘as is’ for visual assessment and the other half was cubed for odour, texture, taste and flavour assessment. The samples were identified by random three-digit codes and presented to judges in a random order. Panellists rated the intensity of each attribute on a five-point structured scale (from 1 = extremely low, to 5 = extremely high) on a paper scorecard and were free to test the cheese cubes as often as they desired. Mineral water and unsalted crackers were used as palate cleansers between samples.

### 2.10. Statistical Analysis

Statistical data analysis was conducted using the JMP Pro 14 program (SAS Institute, Cary, NC, USA). All data were treated with ANOVA (analysis of variance) to analyse the impact of breed (R), ripening (T) and the interaction between breed and ripening (R × T). Regarding the fatty acid profile and sensory analyses, data were analysed considering only the impact of the breed. Sample means were assessed by HSD Tukey’s test and differences were considered significant for *p* < 0.05. Data were reported as least square means ± pooled standard error of the mean (SEM).

## 3. Results

### 3.1. Fatty Acid Profile of Milk and Cheese

First, we analysed the fatty acid profile of the milk and cheese of the two breeds, Teramana and Saanen goats. As observed in Table 1, the fatty acid profiles were different for the two breeds’ milk samples. The main changes were the greater presence of stearic (C18:0, *p* < 0.01), vaccenic (C18:1 trans-11, *p* < 0.01) and linolenic (C18:3, *p* < 0.01) acids, monounsaturated fatty acid (MUFA, *p* < 0.01) and conjugated linoleic acids (CLA, *p* < 0.001) in the Teramana breed. Furthermore, it was possible to observe a greater presence of myristic acid (C14:0, *p* < 0.01) and saturated fatty acid (SFA, *p* < 0.05) in the Saanen breed. As observed in milk, the fatty acids profile of the cheese is very similar and was influenced by the breed (Table 2). In particular, major changes were observed regarding the greater presence of stearic (C18:0, *p* < 0.001), vaccenic (C18:1 trans-11, *p* < 0.001) and linolenic (C18:3, *p* < 0.01) acids, as well as polyunsaturated fatty acid (PUFA, *p* < 0.01) and conjugated linoleic acid (CLA, *p* < 0.001) in the Teramana breed. At the same time, it was possible to observe a greater presence of myristic acid (C14:0, *p* < 0.001) and saturated fatty acid (SFA, *p* < 0.05) in the Saanen breed.

### 3.2. Western Blot Analysis of Δ^9^-Desaturase and Fatty Acid Synthase

**Figure 1:**
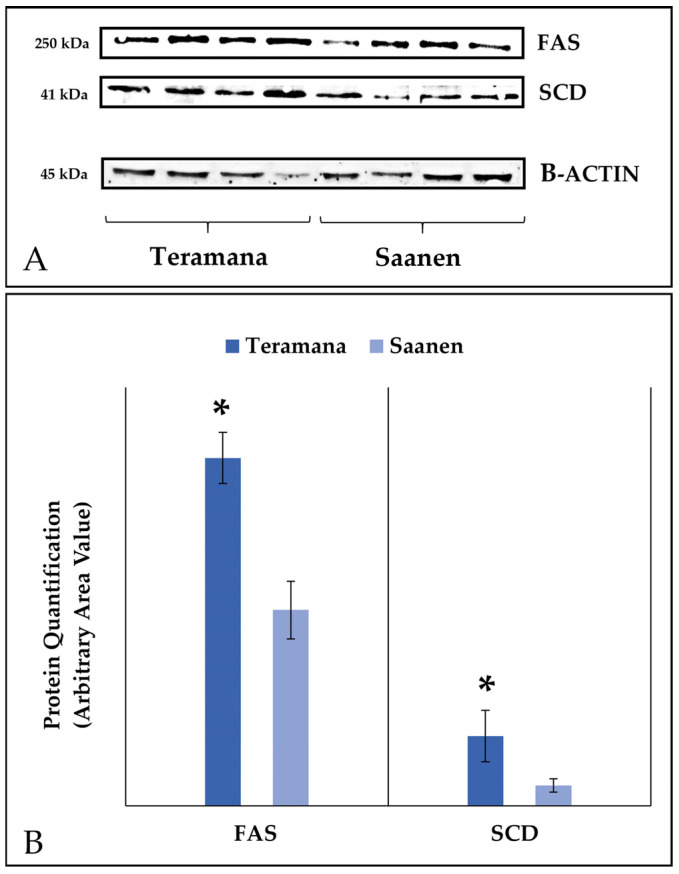
Western blot analysis of SCD and FAS. (**A**) Representative image of WB of SCD and FAS in somatic cells of milk from Teramana and Saanen goat. (**B**) Densitometric values normalised on β-actin expression. Data are reported as area percentage ± S.D. * *p* < 0.05. (*n* = 3).

To investigate variations in the fatty acid profile, we carried out a Western blot analysis on somatic cells to identify Δ^9^-desaturase (SCD) and fatty acid synthase (FAS). Specific antibodies were used to verify the presence of SCD and FAS in somatic cells isolated in milk samples (Figure 1). As represented in Figure 1, a significant increase in SCD and FAS was observed in Teramana milk compared to Saanen milk (*p* < 0.05).

### 3.3. Cheese Moisture, Total Lipids

In order to obtain a detailed knowledge of the differences in production between the breeds, the cheeses, ripened for 0, 30 and 60 days, were analysed for moisture and total fat content. As shown in Table 2, for moisture content, the variation was identified as an effect of both breed and ripening time (*p* < 0.001). The same result was obtained for the total fat content (calculated on a DM basis), which shows significant changes in both breed and ripening time, with an increase in total fat in cheese with ripening times T30 and T60 compared to fresh cheese (T0) (*p* < 0.001). 

### 3.4. Cheese Protein Profile and Proteolysis

**Figure 2:**
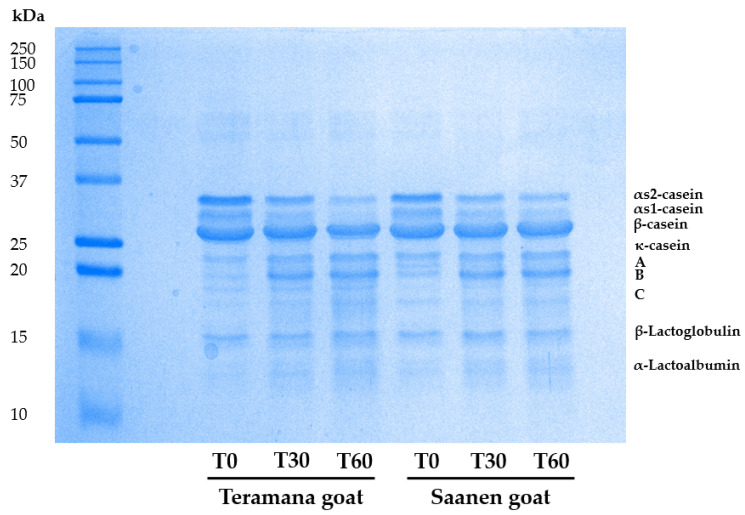
A representative image of SDS-PAGE analysis of cheeses at 0 (T0), 30 (T30) and 60 (T60) days of ripening, obtained from the milk of Teramana and Saanen goats. (*n* = 3).

The cheese protein profile was assessed using an electrophoretic approach under denaturing and reducing conditions (Figure 2); the variations observed for casein and band A and band C, resulting as products from the proteolysis, were dependent on the maturing period and not on the breed. For αs2-casein, αs1-casein, β-casein, κ-casein, band A and band C, there is a statistical significance (*p* < 0.001), while for the whey proteins (β-lactoglobulin and α-lactalbumin) no significant differences were detectable (Table 3).

### 3.5. Identification of Volatile Compounds in Cheese

The analysis of VOCs identified compounds from four chemical families: carboxylic acids, ketones, aldehydes and methyl esters (Table 4). The behaviour of these classes of compounds was significantly influenced by the maturing period in the two breeds. Carboxylic acids showed higher concentrations in Teramana samples at 60 days of ripening (Octanoic acid, *p* < 0.05), a condition that appeared to be closely associated with the concomitant and significant reduction of ketones (2-Heptanone and 2-Nonanone, *p* < 0.01) and methyl esters (Hexadecanoic acid methyl ester, *p* < 0.05), which are more frequently expressed in the Saanen breed. Meanwhile, aldehydes do not show a significant difference between the two breeds.

### 3.6. Confocal Microscopy Image of Cheese

**Figure 3:**
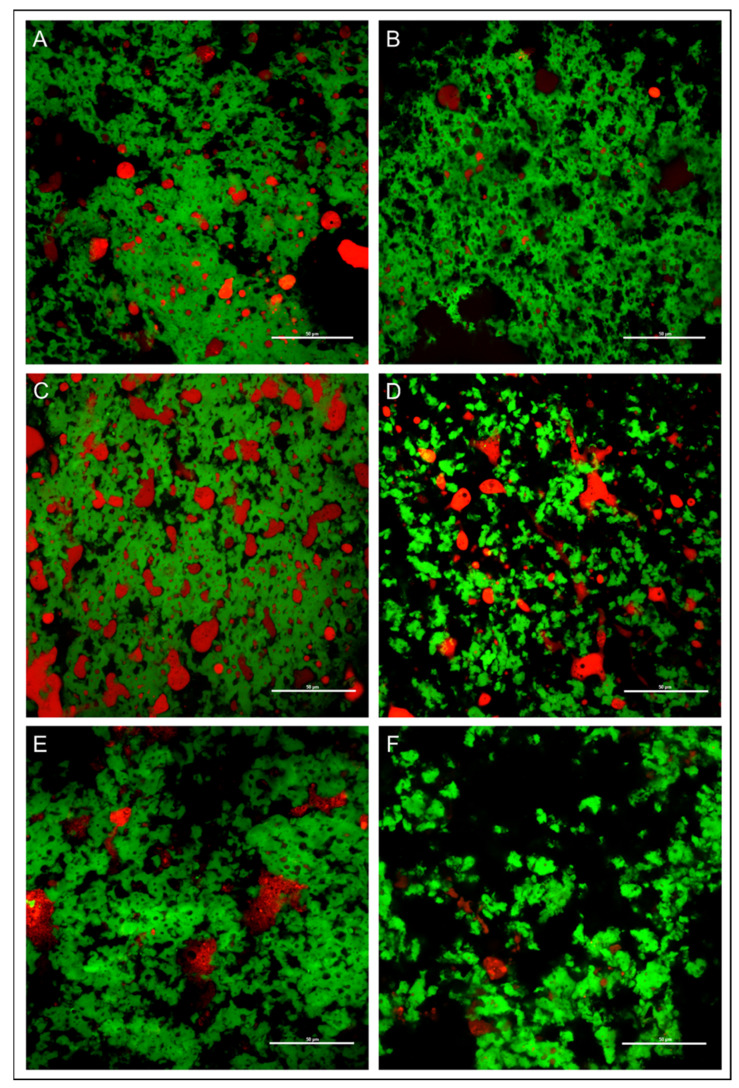
Microstructure of cheese. (**A**,**C**,**E**) Confocal microscopy image of cheese from Teramana goat milk; (**B**,**D**,**F**) cheese from Saanen goat milk. Samples were ripened for 0, 30, 60 days. The Nile Red stained fat appears red and the fast green FCF stained protein appears green in these images.

The structure of the samples was also analysed under the confocal microscope after 0, 30 and 60 days of ripening (see Figure 3). The images show a continuous protein network permeated by fat, more organised and compact in cheese samples produced with milk from Teramo goats compared to samples from Saanen goats.

### 3.7. Physical and Sensorial Properties of Cheese

Regarding the physical parameters, cheese has been characterised by colour and rheological profile. In regard to the colour, the breed and the ripening time seem to have significantly influenced the brightness (L*), the yellow chromaticity (b*) and YI (Table 5, *p* < 0.001), the values of which were lower in T30 and T60 cheese in both of the two breeds. 

**Figure 4:**
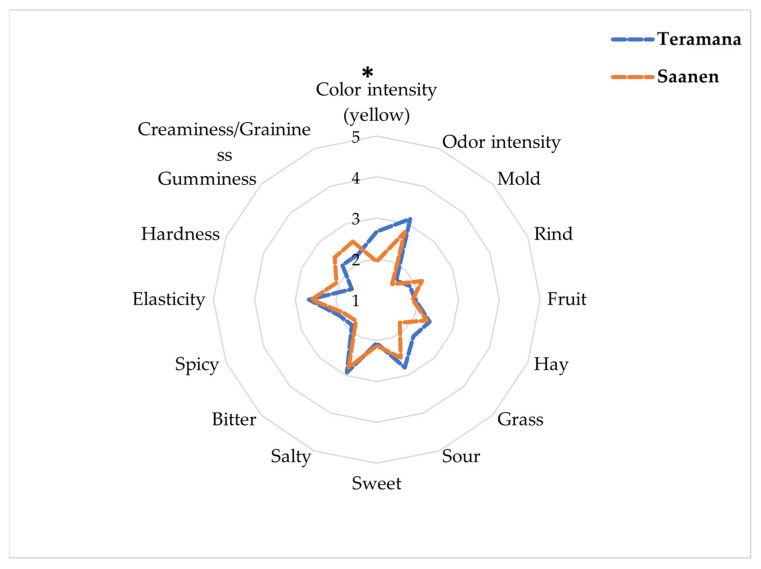
Graphic representation of the sensory evaluation by quantitative descriptive analysis at 60 days of ripening obtained from the milk of two goat breeds: Teramana and Saanen. *: *p* < 0.05.

The rheological profile was evaluated for hardness, cohesivity and gumminess of cheeses, which increased along with ripening time (Table 5). Only the ripening time influenced the texture profile of the cheeses, with a significant increase in hardness (*p* < 0.001) and gumminess (*p* < 0.001). From this point of view, no variation was highlighted in regard to the cohesivity. Upon completion of the instrumental analyses, a sensory analysis was carried out after the recruitment of untrained panellists. A quantitative descriptive analysis was conducted on cheeses at the end of ripening (T60), therefore ready for consumption, by evaluating 16 quality attributes. The results are reported in Figure 4. The untrained panellists found Teramana’s cheese to have a higher colour intensity than Saanen’s cheese (*p* < 0.05). The results agreed with the previous analytical results. 

## 4. Discussion

The milk from Teramana goat, an indigenous breed from central Italy, was used in this study to produce and characterise the chemical and nutritional qualities of the cheese.

Intriguing variations in the relative quantity of the fatty acids composition of both milk and cheese were discovered, and the same variations were found in both products. The Saanen goat’s milk and cheese samples were found to be richer in SFA, a finding that was fully supported by greater levels of myristic (C14:0) and palmitic acids (C16:0). There is little doubt that the most logical explanation for these disparities should be explored endogenously. The Teramana goats had a greater desaturation index for myristoleic (C14:1) and palmitoleic (C16:1) acids; this is deeply related to higher expression levels or simply higher activity levels of the enzymes implicated in these pathways, including SCD [30]. The higher SCD expression in the Teramana goat was confirmed by WB analysis on milk samples. This is further supported, at least in part, by Saanen samples’ lower levels of C18:1 and C16:1. 

Other factors are involved in the metabolism of fatty acids, such as the degree of expression or activity of certain enzymes. In several studies, it has been seen that in rustic breeds [31,32], these enzymes are more expressed. Fatty acid synthase (FAS) is a multifunctional enzyme responsible for the de novo production of palmitate from acetyl-CoA, malonyl-CoA and NADPH [33]. It is conceivable that the Teramana goat exhibits a higher level of this enzyme’s constitutive expression, which would have a substantial effect on the production of FA. In fact, in Teramana samples, the increased concentration of FAS has been confirmed by WB. In addition, since Teramana samples were found to be higher in stearic acid (C18:0), we could speculate that the lower levels of C16:0 in these samples are a result of elongase-6 using C16:0 as a substrate more frequently (elongase of the long chain fatty acid family 6, ELOVL6). The extension of the palmitate stearate chain is catalysed by this microsomal enzyme, which is ubiquitous in practically all mammalian tissues and influences the fatty acid content of tissues [34]. 

It was also possible to highlight a significant increase in the concentration of linoleic acid conjugates (CLA), whose production is correlated to the activity of SCD. The beginning of this process is given by vaccenic acid (C18:1 trans-11), which, with the activity of the enzyme SCD, converts vaccenic acid into CLA [35] in the mammary gland. 

Stearic acid (C18:0) represents the final product of rumen biohydrogenation, leading to an increased concentration of this fatty acid. As with the enzyme FAS, also for SCD, it is conceivable that Teramana goats may have higher expression levels, validated by WB. On the other hand, the presence of more vaccenic acid would promote the increased conversion and activity of the enzyme, obtaining a greater production of CLA and confirmed by a greater desaturation index of CLA. Given its correlation with lower risks for coronary heart disease and cancer growth, this condition is typically linked to an improvement in the nutritional qualities of dietary products [36].

Then, the volatile profile in cheese was analysed to assess in detail the differences between breeds. The results showed four different chemical families: carboxylic acids, ketones, aldehydes and methyl esters. Carboxylic acids are the most prevalent family in the samples studied, derived from the lipolysis of the triglycerides by several enzymes from microbial, milk and added rennet pastes [37]. Carboxylic acids contribute, on the one hand, to the formation of the cheese flavour directly and, on the other hand, to the indirect formation of methyl ketones, secondary alcohols, aldehydes, lactones and esters [38]. Noteworthy, octanoic acid is significantly higher in cheeses made from Teramana goat milk than in those made from Saanen goat milk. 

In this regard, ketones were detected in cheese samples. In detail, 2-Heptanone and 2-Nonanone were significantly higher in cheeses made from Saanen goat milk. These compounds are correlated with strong, unattractive aromatic notes that, due to their extremely low threshold, can be detected despite the presence of low concentrations [39]. They can also be further converted to secondary alcohols by moulds (such as *Penicillium* spp.) [37]. Since cheese is a very reducing environment, unsaturated fatty acids may be involved in autoxidation, producing aldehydes that are characterised by “green grass-like” aromas, such as nonanal [14], found in samples of cheese made from Teramana goat milk. 

The SDS-PAGE was carried out to determine the entity of the various protein fractions in cheeses during maturation and their total protein content to monitor the potential proteolytic process. As caseins are the primary protein source in milk and are involved in enzymatic coagulation, they are positively correlated with cheese production [39]. The level of degradation affects the creation of their specific textures and plays a significant role in the development of flavours, since peptides and amino acids are produced [40]. Casein is particularly significant because it contributes to cheese yield, curd firmness, syneresis rate, moisture retention, and ultimately cheese quality by creating the structural matrix of the curd that holds fat and moisture [41,42]. The SDS-PAGE patterns of cheese showed a great proteolytic activity of αs2- CN and αs1-CN and a high presence of β-CN and κ- CN. Caseins were all susceptible to proteolysis, but to varying degrees. In general, αs-CN was more susceptible than β-CN and κ- CN. After 30 days of ripening, the residual content of these caseins was lower. β-CN and κ- CN were more resistant to proteolysis than αs-CN, particularly after 30 days. These findings are consistent with previous research [42], which found that β-CN degradation progressed over a longer period of ripening. Holt et al. [43] and Prieto et al. [44] observed that the β-CN had a lower degree of hydrolysis than the αs-casein during ripening due to low plasmin activity and its resistance to chymosin. On the other hand, it has been shown that the structure and arrangement of cheese proteins, which control the accessibility of proteolytic enzymes to the cheese substrates, substantially influence the rate of protein degradation [45].

The colour analysis evaluation evidenced a significant reduction in lightness in both samples at the end of the ripening period (T60). As previously reported by Eissa et al. [46], the lightness in a dairy product can be directly related to the moisture content. Since our study has shown a greater loss of humidity by the cheese after 60 days of cheesemaking, it is plausible that this could represent the most obvious key reading of the observed finding. In addition, both exhibit noticeably larger values of the chromatic coordinate b*, which attests to the dairy product’s propensity to assume a lighter totality. The literature links this type of fluctuation to the existence of water-soluble or fat-soluble metabolites that can be taken by ruminants along with their feed and released into the milk through the mammary gland, which may partially help to explain this observation. As an example, larger values of the b* parameter in cheese result from the presence of fat-soluble vitamins, such as retinol and xanthophyll, which cause the cheese to appear yellower and lighter [47]. Considering this, a greater presence of these compounds in the diet is hypothesised.

The various proteolytic pathways could oversee the changes in the texture of the cheeses and are regarded by consumers as indicators of the overall quality of the final product. As the metrics are invariably tight in relation to the amount of water and the makeup of the fat and proteins that define the food matrix, and since their interpretation is not always straightforward, a TPA test was conducted to measure the many textural parameters [48]. Since the force–displacement curves showed that no fracture occurred within this deformation extent, the samples in this study were compressed to 20% of their height rather than the initial 25% compression extent in TPA [27]. Casiraghi et al. [49] also investigated the same deformation and discovered that it had a greater link with sensory hardness than higher deformations. In this study, the Teramana cheeses were characterised by higher hardness than the Saanen ones at the end of ripening time. Considering that the moisture content of these samples decreases, hardness increase during ripening could be related to the moisture decrease, with water as the major plasticizer in food [50,51]. The same thing happens for gumminess, but this could be explained by proteolytic phenomena. This may relate to the changes occurring in the matrix of caseins, since the α-caseins presented significant changes in the same period of maturation. These results show a positive correlation between hardness and α-caseins [52]. 

The findings of the TPA test were enhanced by analysing the cheese samples under confocal microscopy. Cheese microstructure is deeply related to sensory and nutritional properties. Several variables have an effect on the microscopic organisation of cheese, for instance, renneting pH, the temperature during coagulation and calcium content [53]. Our results show a continuous protein network permeated by fat, being more organised and compact in cheese samples produced with milk from Teramana goats compared to milk from Saanen goats, confirming the data from the TPA test.

Finally, the results showed clear differences between the breeds. Thus, it was deemed appropriate to carry out a panel test in order to clarify the actual degree of acceptability of the experimental dairy products by the consumers.

The sensory analysis results confirm the information given by the colour analysis, with Teramana cheese presenting a yellower colour than the Saanen cheese. The hardness and saltiness of the cheese (*p* = 0.051) confirm some information given by the texture analysis, both influenced by less moisture and proteolytic activity since the final products formed during proteolytic phenomena could act as saltiness enhancers [54]. Increased levels of hardness, saltiness and yellowness may increase the product’s acceptability by cheese customers who value a flavourful cheese. As a result, this could make Teramana cheese a great candidate in the dairy market.

## 5. Conclusions

The current study gathers important data for the valorisation of milk and cheese derived from Teramana goats, a local breed of central Italy. Specifically, milk and cheese products were rich in CLA, the consumption of which in animal products is frequently correlated to significant consumer health benefits. Reduced concentrations of compounds such as ketones and esters, and greater concentrations of carboxylic acids would improve the cheese’s oxidative stability during the ripening process. The variations in volatile profile, lipolytic action and technological characteristics were in part confirmed by sensory analyses. The hardness, saltiness and odour intensity of the cheeses increased, indicating an improvement in customer acceptance of the product. Thus, this encourages the manufacture of traditional cheese variations that can fill specific market niches.

## Figures and Tables

**Table 1:** Fatty acid composition of milk and fresh cheese (T0) obtained from two goat breeds: Teramana and Saanen.

	Milk				Cheese			
	Teramana	Saanen	SEM	*p*-Value	Teramana	Saanen	SEM	*p*-Value
C4:0	1.75	1.65	0.16	ns	2.13	1.33	0.05	ns
C6:0	2.38	2.23	0.20	ns	2.57	1.94	0.09	ns
C8:0	3.22	3.02	0.26	ns	3.22	2.86	0.22	ns
C10:0	11.21	11.20	0.85	ns	10.79	11.99	3.85	ns
C12:0	4.92	5.21	0.29	ns	3.83	5.95	0.56	ns
C14:0	9.84 ^b^	11.02 ^a^	0.24	0.01	9.51 ^b^	11.41 ^a^	0.68	0.001
C15:0	1.23	1.06	0.01	ns	1.00	1.10	0.01	ns
C16:0	26.42 ^b^	28.77 ^a^	0.44	0.01	28.89 ^b^	29.28 ^a^	0.91	0.001
C18:0	11.00 ^a^	9.83 ^b^	0.52	0.01	13.58 ^a^	11.75 ^b^	0.96	0.001
C20:0	0.33	0.23	0.01	ns	0.37	0.21	0.01	ns
C14:1	0.43	0.40	0.01	ns	0.43	0.40	0.01	ns
C16:1	0.35	0.26	0.02	ns	0.96	0.74	0.01	ns
C18:1,t11	1.57 ^a^	1.10 ^b^	0.06	0.01	1.53 ^a^	0.87 ^b^	0.02	0.001
C18:1,c9	17.48	16.33	0.73	ns	14.77	14.65	2.98	ns
C18:1,c11	0.08	0.11	0.02	ns	0.16	0.29	0.01	ns
C18:2	1.22	1.31	0.04	ns	1.88	1.31	0.06	ns
C18:3	1.06 ^a^	0.93 ^b^	0.04	0.01	1.04 ^a^	0.89 ^b^	0.01	0.01
SFA	72.50 ^b^	74.85 ^a^	0.97	0.05	73.44 ^b^	76.75 ^a^	3.09	0.05
MUFA	19.82 ^a^	17.90 ^b^	0.79	0.01	19.54	17.20	3.81	ns
PUFA	4.07	3.89	0.14	ns	3.52 ^a^	2.81 ^b^	0.10	0.01
CLA	1.80 ^a^	1.65 ^b^	0.07	0.001	1.79 ^a^	1.29 ^b^	0.01	0.001
OTHERS	3.71	3.69	0.09	ns	1.59	1.52	0.01	ns
DI C14	4.19 ^a^	3.50 ^b^	0.12	0.01	4.35 ^a^	2.95 ^b^	0.20	0.01
DI C16	0.94 ^a^	0.90 ^b^	0.26	0.01	2.90 ^a^	2.47 ^b^	0.13	0.01
DI C18	61.38	61.99	0.45	ns	52.65	58.48	0.44	ns
DI CLA	44.95 ^a^	40.00 ^b^	2.25	0.01	55.78 ^a^	44.34 ^b^	3.31	0.01

All data are reported as least square means the percentage of total fatty acids. Different letters in the same row indicate significant differences. SEM: pooled standard error of the mean; ns: not significant. CLA = Conjugated Linoleic Acids; SFA = Saturated Fatty Acids; MUFA = Monounsaturated Fatty Acids; PUFA = Polyunsaturated Fatty Acids; DI = Desaturation Index.

**Table 2:** Chemical composition of cheese at 0 (T0), 30 (T30), and 60 (T60) days of ripening, obtained from the milk of two goat breeds: Teramana and Saanen.

	T0		T30		T60		SEM	*p*-Value		
	Teramana	Saanen	Teramana	Saanen	Teramana	Saanen		R	T	R × T
Dry Matter (DM)	52.06 ^d^	40.37 ^e^	73.48 ^b^	60.08 ^c^	77.82 ^a^	72.25 ^b^	4.00	0.001	0.001	0.001
Lipids %	23.35 ^b^	14.54 ^c^	28.89 ^a^	16.86 ^c^	27.77 ^ab^	18.28 ^c^	6.51	0.001	0.001	ns

All data are reported on a dry matter basis (DM). Different letters in the same row indicate significant differences. SEM: pooled standard error of the mean. R: breed; T: ripening; ns: not significant.

**Table 3:** Densitometric analysis of SDS-PAGE protein bands (Figure 1) of cheese at 0 (T0), 30 (T30), and 60 (T60) days of ripening, obtained from the milk of goat breeds: Teramana and Saanen.

	T0		T30		T60		SEM	*p*-Value		
	Teramana	Saanen	Teramana	Saanen	Teramana	Saanen		R	T	R × T
αs2-casein	28.18 ^a^	24.00 ^b^	17.91 ^c^	14.11 ^d^	8.89 ^e^	9.89 ^e^	2.74	0.01	0.001	0.01
αs1-casein	9.19 ^ab^	10.58 ^a^	7.01 ^abc^	5.28 ^bc^	3.28 ^c^	3.77 ^c^	3.98	ns	0.001	ns
β-casein	33.19 ^b^	33.12 ^b^	39.14 ^ab^	38.73 ^ab^	37.77 ^ab^	40.20 ^a^	8.55	ns	0.001	ns
κ-casein	7.00 ^b^	8.00 ^b^	7.30 ^b^	8.65 ^ab^	12.16 ^a^	10.02 ^ab^	2.89	ns	0.001	ns
A	3.30 ^b^	3.63 ^b^	12.60 ^a^	10.16 ^a^	13.51 ^a^	8.66 ^a^	4.62	ns	0.001	ns
B	3.29	2.54	2.55	4.46	1.80	8.29	4.24	ns	ns	ns
C	1.92 ^b^	3.78 ^b^	2.86 ^b^	2.66 ^b^	5.85 ^a^	4.07 ^a^	0.64	ns	0.001	ns
β-lactoglobulin	11.23	11.80	8.19	14.01	12.69	11.46	3.18	ns	ns	ns
α-lactoalbumin	2.65	2.50	2.39	1.89	4.00	3.59	1.56	ns	ns	ns

All data are reported as relative least square means, with the percentage of the total protein content calculated in the reference lane. Different letters in the same row indicate significant differences. SEM: pooled standard error of the mean. R: breed; T: ripening; ns: not significant.

**Table 4:** The main classes of volatile compounds detected in cheese at 60 (T60) days of ripening were obtained from the milk of Teramana and Saanen breeds.

	Teramana	Saanen	SEM	*p*-Value
Aldehydes				
Nonanal	0.26	nd	0.03	ns
Carboxylic Acids				
Butanoic acid	2.21	5.31	1.48	ns
Hexanoic acid	28.09	21.36	1.60	ns
Octanoic acid	35.63 ^a^	31.78 ^b^	2.70	0.05
Nonanoic acid	0.40	0.18	0.07	ns
Decanoic acid	24.23	21.47	2.36	ns
Dodecanoic acid	0.82	0.84	0.03	ns
Ketones				
2-Heptanone	0.36 ^b^	7.48 ^a^	0.69	0.01
2-Nonanone	6.52 ^b^	6.96 ^a^	2.86	0.05
2,7-Octanedione	0.26	0.17	0.05	ns
Methyl ester				
Hexadecanoic acid, methyl ester	0.05 ^b^	0.27 ^a^	0.01	0.05
Others	0.18	0.13	0.01	ns

All data are reported as least square means. Different letters in the same row indicate significant differences. SEM: pooled standard error of the mean. ns: not significant; nd: not detectable.

**Table 5:** Physical properties of cheese at 0 (T0), 30 (T30), and 60 (T60) days of ripening, obtained from the milk of goat breeds: Teramana and Saanen.

	T0		T30		T60		SEM	*p*-Value		
	Teramana	Saanen	Teramana	Saanen	Teramana	Saanen		R	T	R × T
Color										
L*	92.12 ^a^	88.10 ^b^	82.36 ^c^	81.07 ^c^	83.82 ^c^	81.54 ^c^	3.82	0.001	0.001	ns
b*	10.46 ^ab^	7.48 ^c^	12.07 ^a^	9.10 ^bc^	11.44 ^a^	9.01 ^bc^	0.95	0.001	0.001	ns
YI	16.23 ^bc^	12.16 ^d^	21.02 ^a^	16.04 ^c^	19.49 ^ab^	15.78 ^c^	3.45	0.001	0.001	ns
Texture										
Hardness	4.03 ^b^	1.49 ^b^	20.39 ^a^	22.31 ^a^	25.27 ^a^	21.24 ^a^	25.01	ns	0.001	ns
Cohesivity	0.97	0.99	0.99	0.99	0.99	0.98	0.10	ns	ns	ns
Gumminess	3.92 ^b^	1.48 ^b^	20.34 ^a^	22.25 ^a^	25.21 ^a^	21.06 ^a^	25.15	ns	0.001	ns

All data are reported as least square means. L*: lightness; b*: yellow/blue chromaticity; YI: 142.86^.^(b*/L*). Different letters in the same row indicate significant differences. SEM: pooled standard error of the mean. R: breed; T: ripening; ns: not significant.

## Data Availability

The data presented in this study are available on reasonable request from the corresponding author.

## Supplementary Materials

The following supporting information can be downloaded at: https://www.mdpi.com/article/10.3390/ani13081344/s1. Supplementary Materials: representative images of Teramana goats and cheeses at different ripening time. Figure S1: A typical goat breed of Teramo, called the Teramana goat. These medium-sized goats have a dark coat (primarily black or dark brown), a long head with white streaks, a straight frontal nasal profile, and the potential for horns in both sexes. Figure S2: Images of Teramana cheeses at 0 (T0, A), 30 (T30, B) and 60 (T60, C) days of ripening.
