# Supplementary material for: New Insight into the Quality Traits of Milk and Cheese from Teramana Goats, a Native Italian Breed
Source: Animals (Basel). 2023 Apr 13;13(8):1344. doi: 10.3390/ani13081344 (PMC10135095; doi:10.3390/ani13081344)
Supplement: Supplementary file 1 [file animals-13-01344-s001.zip › animals-2298767-supplementary.pdf]

Supplementary materials

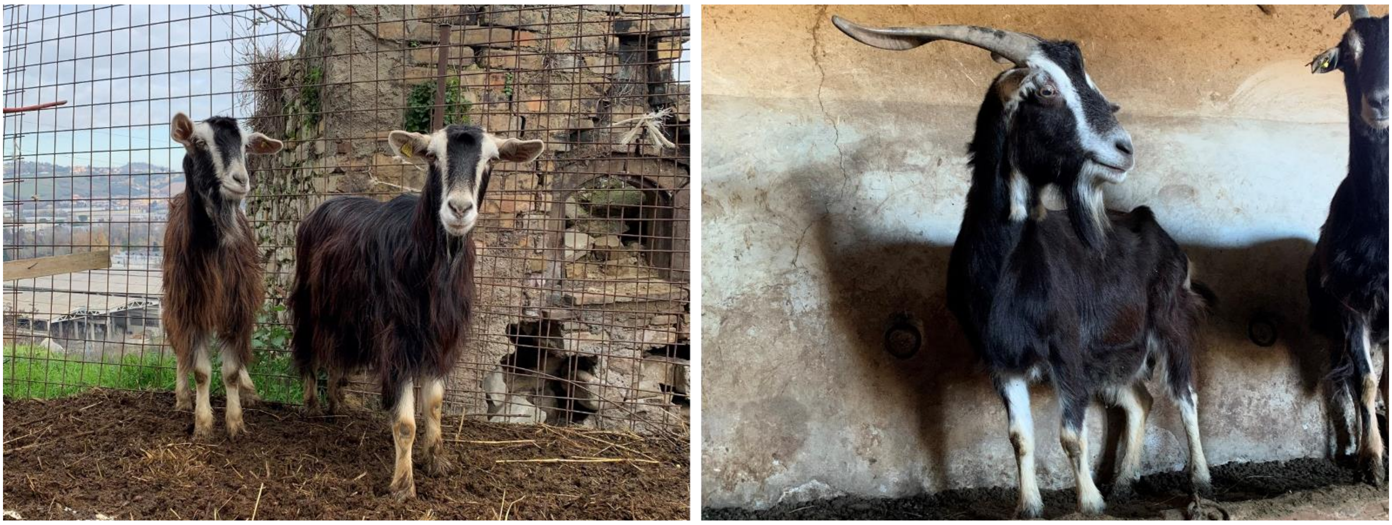

Figure S1: A typical goat breed of Teramo, called the Teramana goat. These medium-sized goats have a dark coat (primarily black or dark brown), a long head with white streaks, a straight frontal nasal profile, and the potential for horns in both sexes.

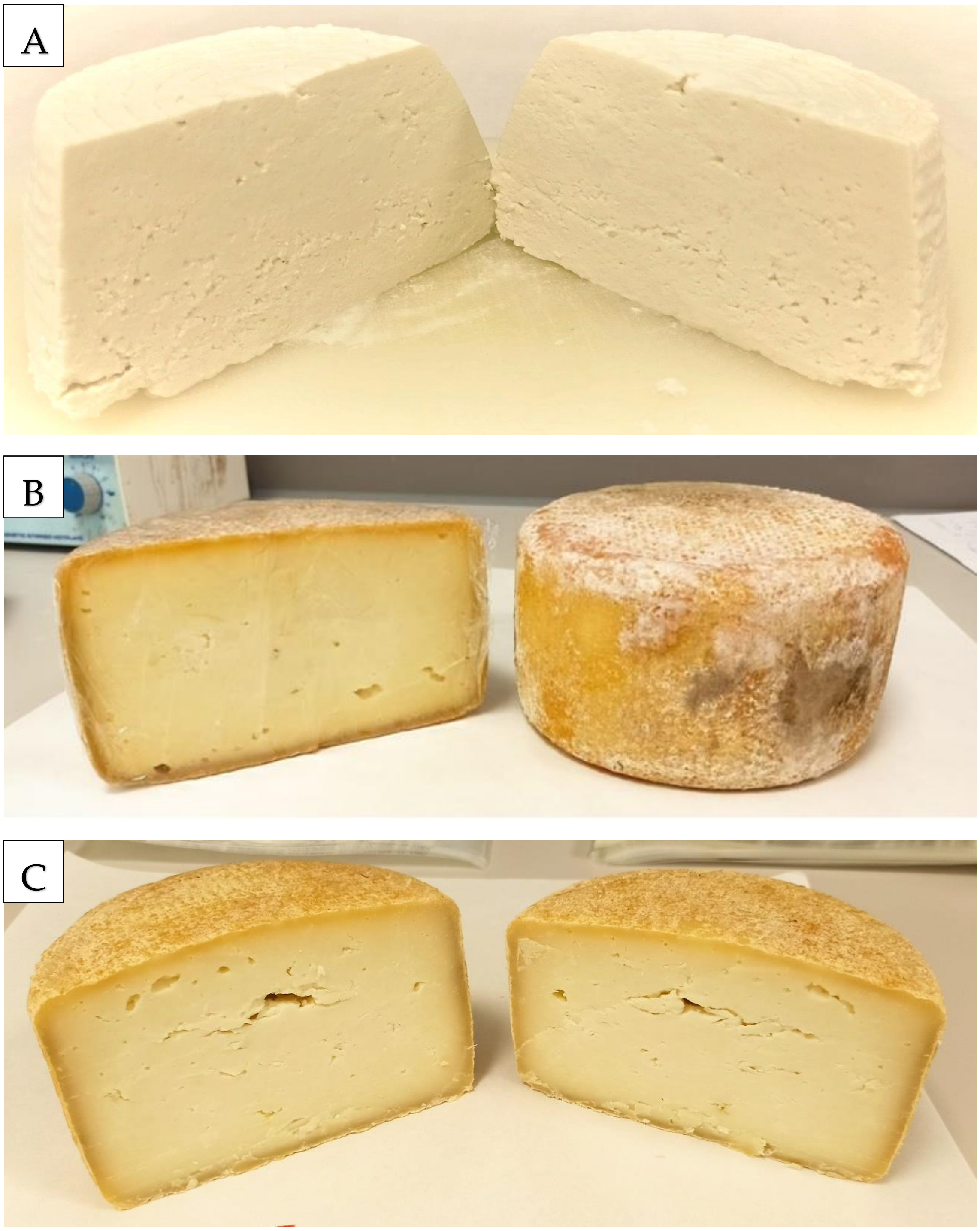

Figure S2: Images of Teramana cheeses at 0 (T0, A), 30 (T30, B) and 60 (T60, C) days of ripening.
